# Tracing the Spread of *Clostridium difficile* Ribotype *027* in Germany Based on Bacterial Genome Sequences

**DOI:** 10.1371/journal.pone.0139811

**Published:** 2015-10-07

**Authors:** Matthias Steglich, Andreas Nitsche, Lutz von Müller, Mathias Herrmann, Thomas A. Kohl, Stefan Niemann, Ulrich Nübel

**Affiliations:** 1 Robert Koch Institute, Department of Infectious Diseases, Division of Nosocomial Pathogens and Antibiotic Resistances, Wernigerode, Germany; 2 Robert Koch Institute, Centre for Biological Threats and Special Pathogens, ZBS 1 Highly Pathogenic Viruses, Berlin, Germany; 3 University of Saarland Medical Centre, Institute of Medical Microbiology and Hygiene, National Consultant Laboratory for *Clostridium difficile*, Homburg, Germany; 4 Research Center Borstel, Molecular Mycobacteriology, Borstel, Germany; 5 German Center for Infection Research (DZIF), Partner Site Borstel, Borstel, Germany; 6 Leibniz Institute DSMZ, Department of Microbial Genome Research, Braunschweig, Germany; 7 German Center for Infection Research (DZIF), Partner Site Hannover-Braunschweig, Braunschweig, Germany; Cleveland Clinic, UNITED STATES

## Abstract

We applied whole-genome sequencing to reconstruct the spatial and temporal dynamics underpinning the expansion of *Clostridium difficile* ribotype *027* in Germany. Based on re-sequencing of genomes from 57 clinical *C*. *difficile* isolates, which had been collected from hospitalized patients at 36 locations throughout Germany between 1990 and 2012, we demonstrate that *C*. *difficile* genomes have accumulated sequence variation sufficiently fast to document the pathogen's spread at a regional scale. We detected both previously described lineages of fluoroquinolone-resistant *C*. *difficile* ribotype *027*, *FQR1* and *FQR2*. Using Bayesian phylogeographic analyses, we show that fluoroquinolone-resistant *C*. *difficile 027* was imported into Germany at least four times, that it had been widely disseminated across multiple federal states even before the first outbreak was noted in 2007, and that it has continued to spread since.

## Introduction

*Clostridium difficile* is the most common cause of healthcare-associated diarrhea in Europe and the USA [1, 2]. In 2009, *C*. *difficile* infection (CDI) was associated with almost 1% of admissions to US hospitals, resulting in a severe burden of morbidity, mortality, and economic costs [3]. In addition, community-associated CDI has been reported, albeit at a lower rate [4–6].

The increase of CDI incidence observed in North America and Europe during the first decade of this century was accompanied by the emergence of a previously uncommon strain of *C*. *difficile* [7, 8], genotyped as PCR ribotype *027*, North American pulsotype *NAP1*, or restriction endonuclease analysis type *BI*, respectively, depending on the genotyping method used (for simplicity, we will refer to this strain as ribotype *027* throughout the remainder of this paper). This epidemic strain caused large, highly publicized outbreaks in hospitals in Canada [9], the US [10], and the UK [11], which were associated with elevated rates of mortality and caused a change of awareness about CDI severity and epidemiology [12]. While conflicting evidence exists regarding the increased virulence of epidemic ribotype *027* (reviewed in [13]), its high-level resistance to fluoroquinolones may have facilitated its spread in healthcare facilities, where this class of antibiotics is widely used [5, 10].

Population genomic analyses recently showed that, in fact, two distinct ribotype *027* lineages, dubbed *FQR1* and *FQR2*, had emerged in North America, after independently acquiring fluoroquinolone resistance in the 1990s [14]. Evidently, both lineages subsequently had spread into Europe on multiple occasions, and either one of them was also found in Australia and South Korea, respectively [14].

In Germany, cases of CDI with epidemic ribotype *027* were first reported in 2007 from a hospital in Stuttgart [15] and from several hospitals around the city of Trier [16]. A nationwide survey indicated that, by 2008, the dissemination of ribotype *027* was mostly restricted to the southwest of Germany [17]. More recent data suggested that ribotype *027* is among the most predominant *C*. *difficile* genotypes in Germany [6] and that its incidence may be increasing [18, 19]. *Clostridium difficile 027* was the most frequently isolated ribotype in a recent prospective survey across 20 European countries; however, 43% of ribotype *027* isolates in that study had been found in samples from Germany alone [20]. Ribotype *027* isolates from Germany commonly are highly resistant to fluoroquinolones [6, 17].

It is unclear, at present, to what extent each of the two internationally dispersed fluoroquinolone-resistant lineages, *FQR1* and *FQR2*, contribute to the burden of CDI in Germany, and what the dynamics of their spread among healthcare institutions may be. In the present study, we generated genome sequence data from 57 *C*. *difficile* ribotype *027* isolates and analysed this data in a Bayesian phylogeography framework to investigate the temporal dynamics of *C*. *difficile* spatial spread in Germany.

## Results and Discussion

### Phylogeny and population structure

Applying Illumina technology, we re-sequenced the genomes from 57 *C*. *difficile* ribotype *027* isolates, 56 of which had been collected from hospitalized patients at 35 locations in Germany between 2007 and 2012, and one isolate had been isolated already in 1990 (S1 Table; sequences were submitted to the European Nucleotide Archive, accession number PRJEB9067). In addition, we included 11 previously published genome sequences representing an international context [14]. Sequencing reads were mapped onto the reference genome sequence from isolate R20291 [14], which had been isolated during a large outbreak in the Stoke Mandeville hospital, UK, in 2005 [11]. After masking variation in mobile genetic elements and repetitive regions, we identified 268 single nucleotide polymorphisms (SNP) in the 3,770,610 basepair core genome (S2 Table). Only a small fraction of mutations (i. e., 13 out of 268 SNPs) were detected in close proximity (≤300 bp) to each other. After removal of these SNPs, the level of homoplasy was very low (homoplasy index, 0.019), suggesting that sequence variation had been generated primarily through mutations and that homologous recombination was rare. The resulting set of 255 SNPs provided the basis for phylogenetic analyses.

**Fig 1:**
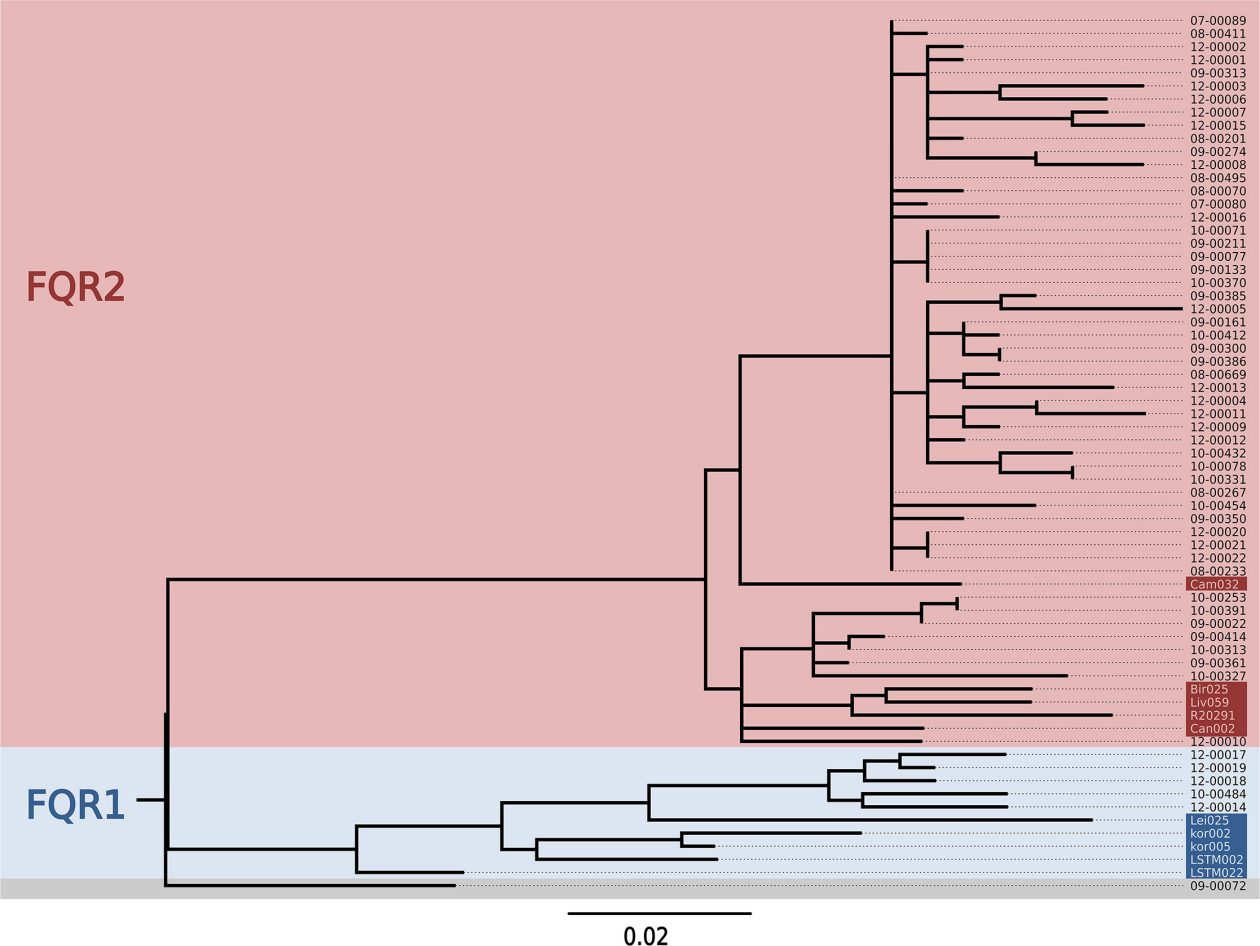
Maximum likelihood phylogenetic tree. The phylogeny of *C*. *difficile* ribotype *027* was reconstructed based on 255 core-genome SNPs. Previously published genome sequences (indicated by shaded isolate names; [14]) were included to enable identification of clades *FQR1* and *FQR2*. The tree was rooted by using the distantly related *BI 3* genome [14] as an outgroup.

A maximum-likelihood phylogenetic tree revealed two major clades (Fig 1). These clades were identified as corresponding to the previously described strains of fluoroquinolone-resistant *C*. *difficile 027*, *FQR1* and *FQR2*, by comparison to several genome sequences from that previous study (Fig 1) [14]. This result demonstrated that both, *FQR1* and *FQR2*, were present in Germany. The majority of isolates in our sample (i. e., 51 out of 57) were related to *FQR2*, however (Fig 1). As reported previously [14], all *FQR1* and *FQR2* isolates carried a missense mutation in their DNA gyrase subunit A gene that rendered them fluoroquinolone resistant (S2 Table). In addition, one isolate from Germany (09–00072), which had been sampled in 1990 and was not resistant to fluoroquinolones, sat at a basal position in the tree and represented the ancestral ribotype *027* population, from which *FQR1* and *FQR2* have emerged (Fig 1, S1 Table) [14]. Bayesian phylogenetic analysis estimated that point mutations had accumulated in the core genome of *C*. *difficile 027* at an average rate of 0.17 mutations per 10^6^ basepairs per year (95% confidence intervals, 0.12 to 0.22 mutations per 10^6^ basepairs per year), which is similar to previous estimates [14, 21]. Based on this calibration, the emergence of *FQR1* was estimated to 1998 (95% confidence intervals, 1988 to 2001) and the emergence of *FQR2* to 1997 (95% confidence intervals, 1987 to 2000), also in accordance with previous estimates [14].

The observed evolutionary rate of *C*. *difficile* is about 10-fold lower than for *Staphylococcus aureus* [22–24] and some other bacteria [25], which limits the discriminatory power of *C*. *difficile* genome sequencing for molecular epidemiology analyses. Accordingly, and due to the stochastic nature of spontaneous mutations, several groups of our isolates had core genome sequences with no detectable differences, despite their temporal or spatial distances. For example, we found identical genomes in five isolates which had been sampled over 19 months (November 2008 to June 2010) in four different hospitals in Southern Germany (Stuttgart, Sindelfingen, Esslingen, Augsburg) (Fig 1, see 09–00077 and related isolates).

### Phylogeographic analyses

For Bayesian phylogeographic inference, we analysed 66 *FQR1* and *FQR2* genomes, 56 of which had been sampled from 35 geographic locations within Germany (S1 Table), and ten of which originated from other countries (including the UK, USA, Canada, Switzerland, Korea, [14]); the latter were included to place the *C*. *difficile 027* population in Germany into an international context. Not unexpectedly, estimates of rates of spatial spread between pairs of these 35 locations yielded insufficient statistical support, because very few data points were available for each individual estimate. Accordingly, discrete Bayes factor tests failed to verify transmissions determined by the Markov chain Monte Carlo analysis (Bayes factors <3 for all individual transmissions, not shown). Therefore, to increase the statistical power of Bayesian phylogeographic analysis, we grouped neighboring locations into regions based on their straight-line distances (S1 Fig). When the number of locations was reduced to 17 regions in Germany, Bayes factors were ≥20 for individual transmissions within Germany (S5 Table), which commonly is considered a significant level of support [26]. Further reduction to 11 regions in Germany yielded Bayes factors >290 for individual transmissions (S5 Table), indicating strong support [26].

### Dynamics of spatial spread

**Fig 2:**
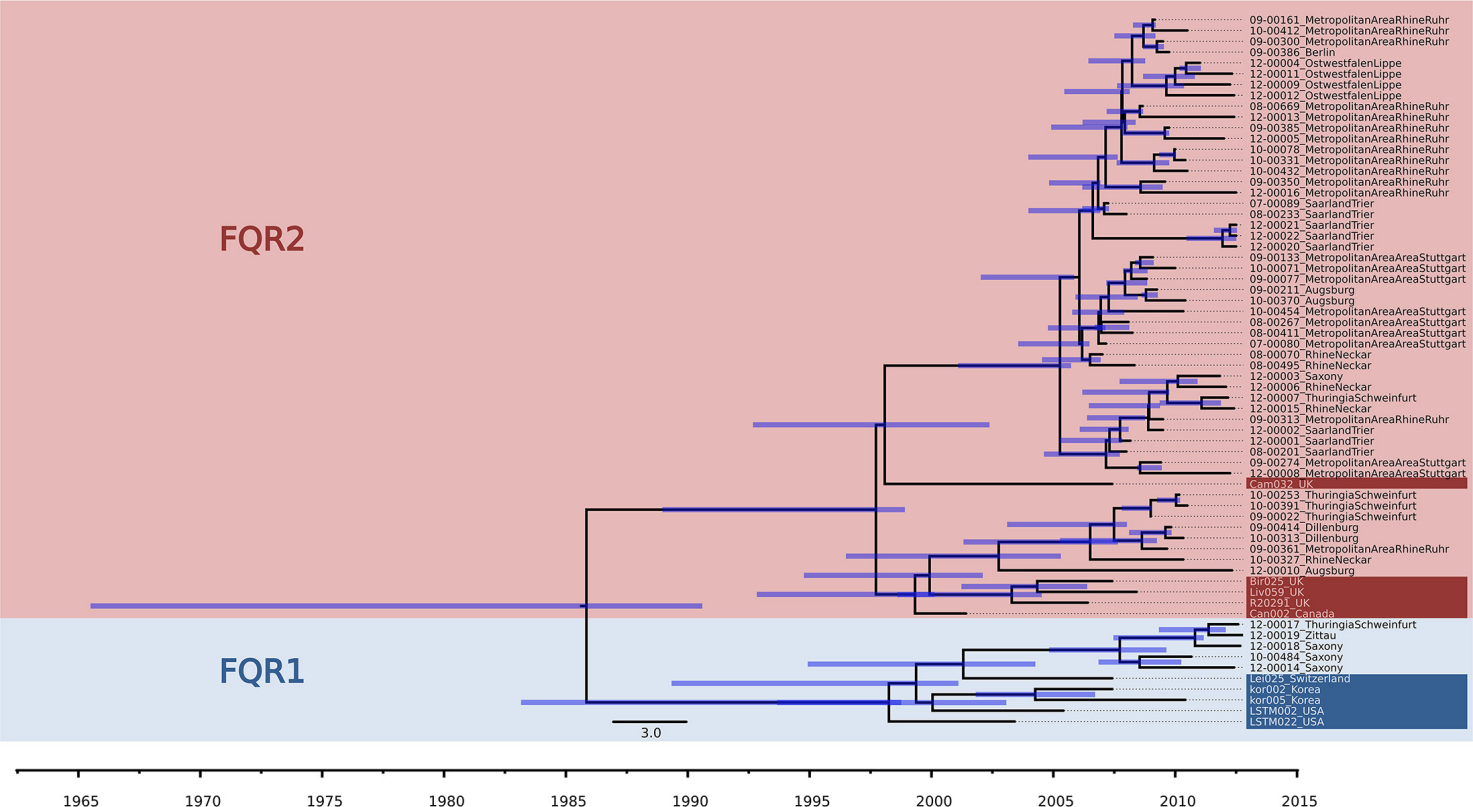
Maximum clade credibility tree based on BEAST analysis of *C*. *difficile* genome sequences. Tips of the tree were constrained by sampling dates, the time scale is shown at the bottom. Blue bars indicate 95% Bayesian credibility intervals of bacterial divergence dates (node heights).

**Fig 3:**
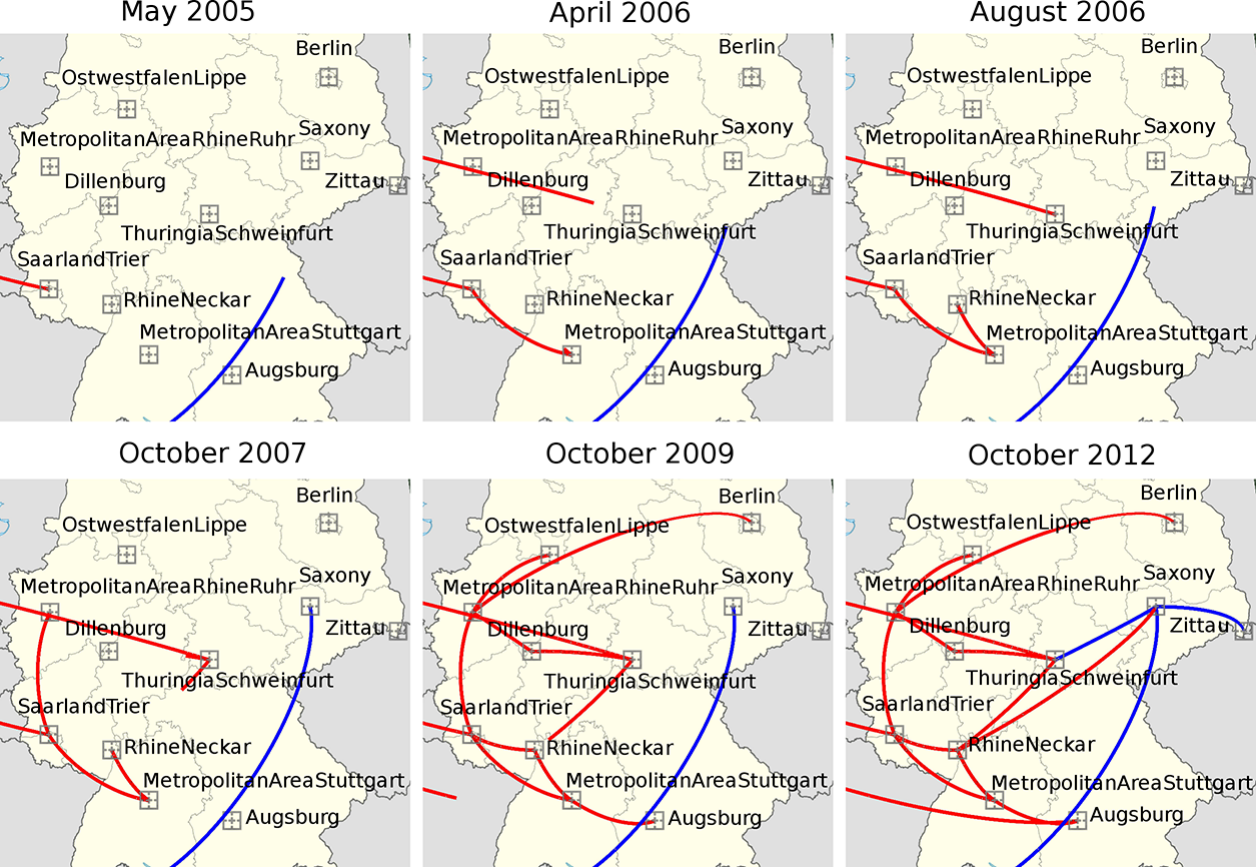
Bayesian reconstruction of the spread of *C*. *difficile 027* in Germany. Squares indicate the centroids of 11 regions and lines indicate the inferred spread of *FQR1* (blue) and *FQR2* (red). Note that, in this discrete analysis, arrival at a region centroid indicates arrival at that region, but trajectories do not represent precise transport routes.

Independent from the level of parameter reduction, our phylogeographic analyses consistently indicated four imports of fluoroquinolone-resistant *C*. *difficile 027* into Germany (Fig 2, Fig 3, S2 Fig and S3 Fig). These four introduction events were inferred from a Bayesian, time-calibrated phylogeny reconstruction (Fig 2) and their directionality was confirmed by Bayesian stochastic search variable selection (BSSVS) (S5 Table) [27, 28]. Lineage *FQR2* was indicated to have been introduced into Southwestern Germany during the first half of 2005 (95% confidence interval, 2001 to end of 2005) (Fig 2, Fig 3), hence some years before it caused a major outbreak affecting several hospitals in the area around the city of Trier [16]. Our analysis considering 11 regions indicated the Saarland/Trier region as the first entry point for *FQR2* (Fig 3), and subsequent spread from there into the Rhein/Ruhr region, the Stuttgart region, and Thuringia (Fig 3). In contrast, our analysis considering 17 regions (i. e., with moderate parameter reduction) suggested the Stuttgart region as the first entry point (S2 Fig), hence this was not clearly resolved. Interestingly, routine surveillance had detected fluoroquinolone-resistant *C*. *difficile 027* in a hospital in Stuttgart in January 2007 [15], simultaneously with the Trier outbreak [16]. In contrast to previous epidemiological analyses, however, our present analyses consistently showed that, by the beginning of 2007, *FQR2* had already been widespread in Germany, affecting four regions in at least four different federal states (Fig 3). Phylogeographic analysis also indicated that the *FQR2* lineage was imported two more times, around 2006 and 2012, apparently into the Thuringia and Augsburg regions, respectively (Fig 3). Interestingly, spread of *FQR2* was restricted mostly to the West of Germany for several years and did not reach Berlin and Saxony prior to 2009 (Fig 3). This finding is in concordance with the distribution of *C*. *difficile 027* in German hospitals prior to 2009 as detected through epidemiological surveillance [17]. In addition, *FQR1* was imported around 2007 (95% HPD, 2004 to 2009), yet its current distribution appears restricted to the East of Germany (i. e., we detected it in the federal states of Sachsen and Thueringen; Fig 3, S1 Table). While available data is limited, it appears from our results and from those of He *et al*. [14] that *FQR1* is less prevalent than *FQR2*, both in Germany and globally, even though they both emerged in North America around the same time. Possibly, *FQR1* is less proliferative than *FQR2*, but the reasons for this difference have not been resolved.

### Limitations

Even though our set of *C*. *difficile* isolates is the most comprehensive collection of *C*. *difficile* ribotype *027* isolates from Germany studied to date, the number of isolates investigated (n = 57) is very small compared to the number of hospitals or to annual numbers of CDI cases [17, 20, 29]. Therefore, the scenario of *C*. *difficile* dissemination and spread we describe may underestimate the complexity of the real situation. Because data available from isolates collected internationally are even more limited [14], we did not attempt to identify sources for introduction into Germany.

### Conclusions

Genome-based phylogenetic analysis indicated that fluoroquinolone-resistant *C*. *difficile* ribotype *027* was imported into Germany at least four times independently. Despite the limited number of isolates included and the comparatively low evolutionary rate of *C*. *difficile*, genome re-sequencing provided conclusive information for Bayesian inference of spread between regions within Germany based on a discrete phylogeographic model. Our analyses indicated that one of the previously described fluoroquinolone-resistant variants of *C*. *difficile 027* (i. e., *FQR2*) has spread within Germany for more than a decade, and the other variant (*FQR1*) had been introduced few years later. At the time when the first outbreak of *C*. *difficile 027* was noticed in Germany in 2007 [16], these newly emerged strains had already been disseminated across hospitals in several federal states, and they have continued to spread since then.

## Methods and Tools

### Isolates

*Clostridium difficile* ribotype *027* isolates (S1 Table) were selected based on results from classical ribotyping [17] or surface layer protein *A* sequence typing [30], respectively, and based on their geographic origin, in order to achieve maximum representation of the pathogen's spatial distribution in Germany. As a result, we included 57 *C*. *difficile* ribotype *027* isolates, collected from hospitalized patients at 36 hospitals in Germany (S1 Table).

### Ethics statement

A formal ethical review process and approval was not required in accordance with article 25, section 1 of the German Infection Protection Act of 2001. All data were analyzed anonymously.

### Raw data

The genomes from *C*. *difficile* isolates were sequenced on HiScan and MiSeq systems (Illumina), producing paired-end reads with lengths of 100 or 250 bases, respectively, to an average >35-fold coverage. Sequencing data were submitted to the European Nucleotide Archive (ENA) and assigned study accession number PRJEB9067. To enable comparisons to previously published genome data [14], sequencing reads from representative *FQR1* and *FQR2* isolates (Fig 1) were downloaded from ENA and included in our analyses. Consensus sequences for individual genomes were determined by applying a read mapping approach combining BWA-SW version 0.7.3a [31] for mapping to a reference genome sequence (acc. no. NC_013316 [32]), SAMtools [33] to handle SAM files (Sequence Alignment/Map) and VarScan (version 2.3) [34] for consensus calling in a customized pipeline framework (VarScan parameters: minimum coverage, 10; minimum average base quality, 20; minimum variant frequency, 0.8; p-value threshold, 0.01). The high-level interpreted language GNU Octave [35] was used to format output files, to analyse the SNP content, and to assemble an alignment of SNPs. Previously published sequencing reads from 11 *C*. *difficile* genomes were included for reference [14](Fig 1).

### Mobile genetic elements and repetitive sequences

Mobile genetic elements may evolve at a different mode and rate than the remainder of the genome, and it is therefore wise to exclude them from phylogenetic analyses. To assort a list of mobile genetic elements in the reference genome, the annotation was screened for keywords and the web server based application PHAST [36] were used to identify mobile genetic elements [32, 37, 38], which were then excluded from phylogenetic analyses (S3 Table). We also excluded CRISPR (S3 Table) and other repetitive DNA sequences, as they tend to create ambiguities in read alignments [39]. Repetitive DNA was detected with the pattern matching engine available in KODON software (Applied Math).

### Phylogenetic analyses

Phylogeny reconstruction is based on the assumption of tree-like evolution, which is violated by homologous recombination that creates mosaic sequences [40]. Therefore we screened our sequences for clustered variation and masked all mutations that had a distance of ≤300 bp from the next mutation. Based on an alignment of core-genome SNPs, PAUP 4b10 (http://paup.csit.fsu.edu/) was used to calculate the homoplasy index, and PhyML implemented in Seaview 4 was used to calculate a maximum-likelihood phylogenetic tree.

### Bayesian inference with BEAST 2.0

To incorporate spatial and temporal components into the phylogeny, the BEAST 2.0 [41] cross-platform was used. It implements a Bayesian framework focused on using strict or relaxed molecular clock models for inference of time-measured phylogenies. As a total re-implementation of the BEAST 1.x software package it provides the option to extend the system with new models via the package system. BEAST 2.0 provides a full Bayesian framework for phylogeographic analysis [27]. The model setup was done following the tutorial "Ancestral reconstruction/discrete phylogeography with BEAST 2.0 (available at http://www.beast2.org/wiki/index.php/Tutorials) with the BEAUti2 xml editor as a part of the BEAST 2.0 framework. We used the beast-classic (BEAST_CLASSIC) add-on and the BEASTii add-on as described in the tutorial. Each sequence was labeled with the year and month of isolate sampling and with ETRS89 (European Terrestrial Reference System 1989) coordinates of sampling locations. Using the HKY model of nucleotide substitution, a strict clock and an uncorrelated relaxed lognormal clock model were tested. The initial clock rate was set to 2 x 10^−7^ substitutions per nucleotide site and per year. On the prior for the nonzero rates (Poisson distribution), the lambda (expected value, variance) was set to 100,693. To test the influence of several tree priors, analyses with different coalescent priors were applied in several runs, under the assumption of a strict clock and an uncorrelated lognormal relaxed clock (S4 Table). For the combined FQR1/FQR2 set without 09–00072 and BI–3 (66 sequences total), 6 x 10^7^ generations was enough to verify proper mixing. Effective sample sizes (ESS) were >200. Trees from every 6,000 generations were sampled. To verify proper priors and to insure that the results are significantly informed by the data, several runs with sampling from priors only were performed and analysed. Samples from three independent Markov chain Monte Carlo runs were combined by applying the LogCombiner software with an exclusion of 15% burn-in [41]. For model comparison, Bayes factors were calculated based on marginal likelihood estimated by using the smoothed harmonic mean estimator [42, 43], as implemented in the Tracer application version v1.6.0.

### Bayesian phylogeography

For discrete phylogeographic analysis, ETRS89 coordinates of sampling locations were incorporated into the model as an additional discrete location trait, applying the BEAST-classic add-on during the model setup with BEAUti 2.0 as described by Lemey *et al* [27]. The Markov chain Monte Carlo analysis was performed as described above, assuming an uncorrelated lognormal relaxed clock and a coalescent constant tree prior. Effective sample sizes (ESS) were >200. Tree files from three independent runs were combined and then processed with the SPREAD 1.0.6 software (Spatial Phylogenetic Reconstruction of Evolutionary Dynamics) [28] to determine the spatial spread in Germany. To identify well-supported transition rates, discrete Bayes factor tests were performed on a BEAST log file with rate indicators (Bayesian stochastic search variable selection (BSSVS) procedure; cutoff, 3) [27]. To increase the statistical power of Bayesian phylogeographic inference, we grouped neighboring locations into regions by using a hierarchical cluster analysis based on straight-line-distances and using the centroid method assuming Euclidean metric (S1 Fig). Setting the level of hierarchy to reduce the number of locations is a compromise to maintain informative spatial resolution and ensure robust statistics at the same time. This way, 11 or 17 regions, respectively, were defined to represent the total of 35 locations in Germany from which isolates had originated, where each region was represented by the coordinates of the centroid of the locations [44]. With these adjustments to the priors, Markov chain Monte Carlo analysis was re-run, and the combined sample data from three independent runs underwent discrete Bayes factor tests to verify the inferred transition rates S5 Table. The visualization of the proliferation dynamics of *C*. *difficile* in time and space was derived from models assuming a relaxed clock.

## Supporting Information

S1 Fig Cluster analysis to group locations into 11 (A) or 17 (B) regions, respectively, based on their distances from each other. (TIF)

S2 Fig Bayesian reconstruction of the spread of *C*. *difficile 027* in Germany, considering 17 regions. Discrete phylogeographic analysis was performed with BEAST 2.0 and SPREAD 1.0.6.
(TIF)

S3 Fig Bayesian reconstruction of the spread of *C*. *difficile 027* in Germany, considering 35 locations. Discrete phylogeographic analysis was performed with BEAST 2.0 and SPREAD 1.0.6.
(TIF)

S1 Table Bacterial isolates. (XLSX)

S2 Table SNPs. (XLSX)

S3 Table Mobile genetic elements. (XLSX)

S4 Table Comparison of BEAST models. Clock rates and Bayes factor tests.
(XLSX)

S5 Table Results of BSSVS analyses. Level of statistical support for individual spreading events.
(XLSX)
