# Supplementary figures and images for: Tracing the Spread of Clostridium difficile Ribotype 027 in Germany Based on Bacterial Genome Sequences
Source: PLoS One. 2015 Oct 7;10(10):e0139811. doi: 10.1371/journal.pone.0139811 (PMC4596877; doi:10.1371/journal.pone.0139811)

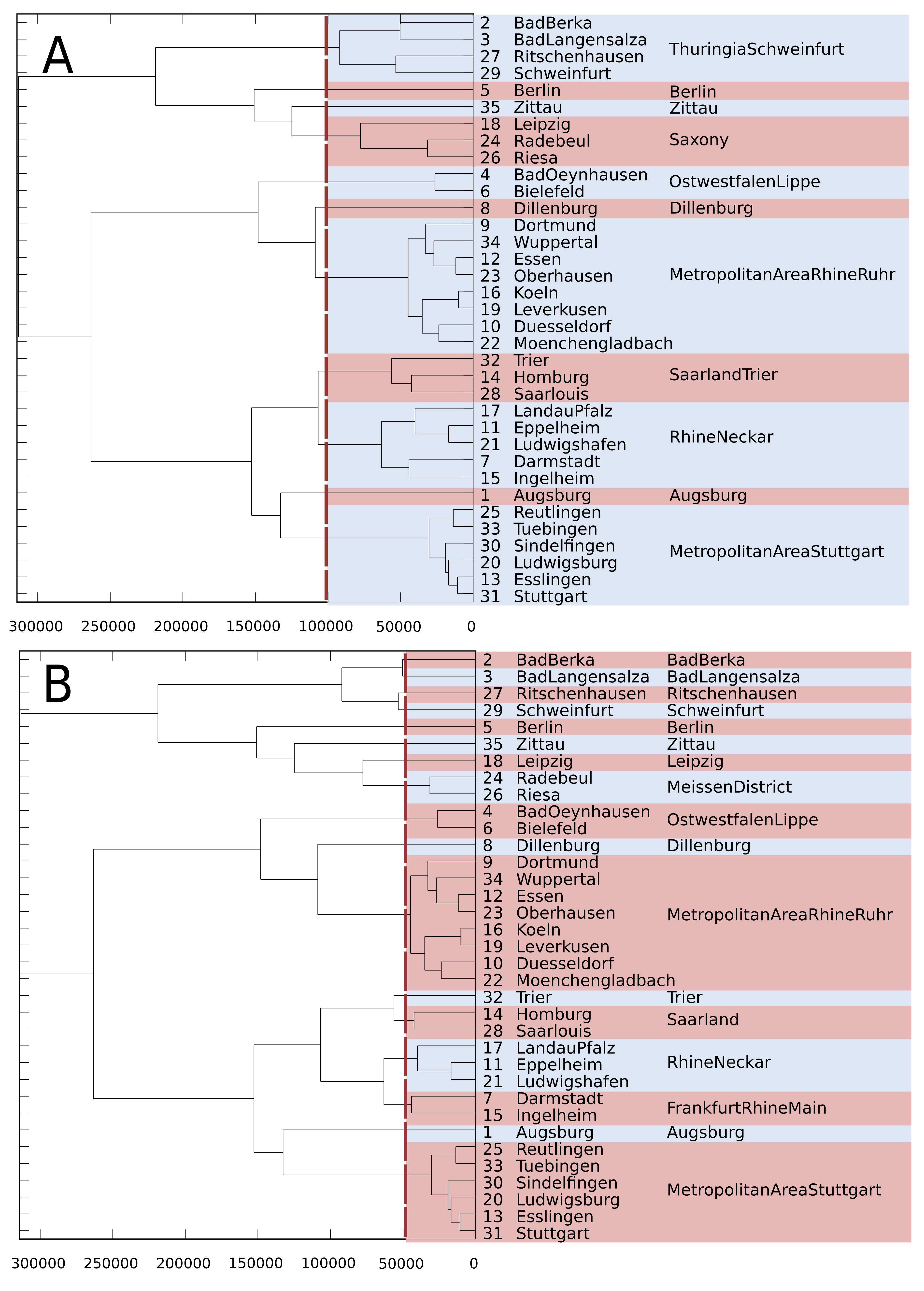

Supplement: S1 Fig — (TIF) [file pone.0139811.s001.tif]

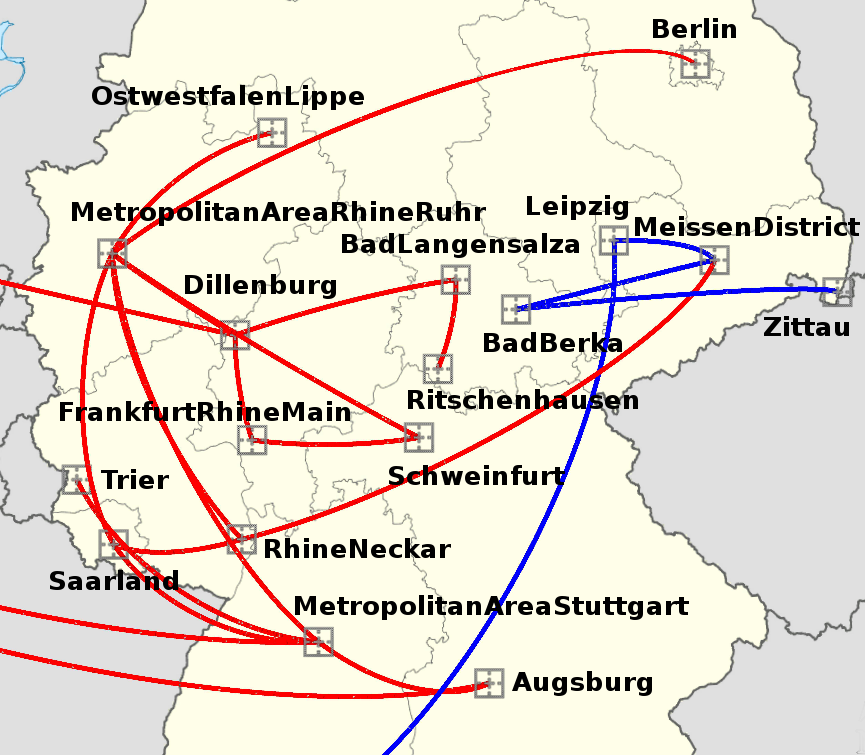

Supplement: S2 Fig — Discrete phylogeographic analysis was performed with BEAST 2.0 and SPREAD 1.0.6. (TIF) [file pone.0139811.s002.tif]

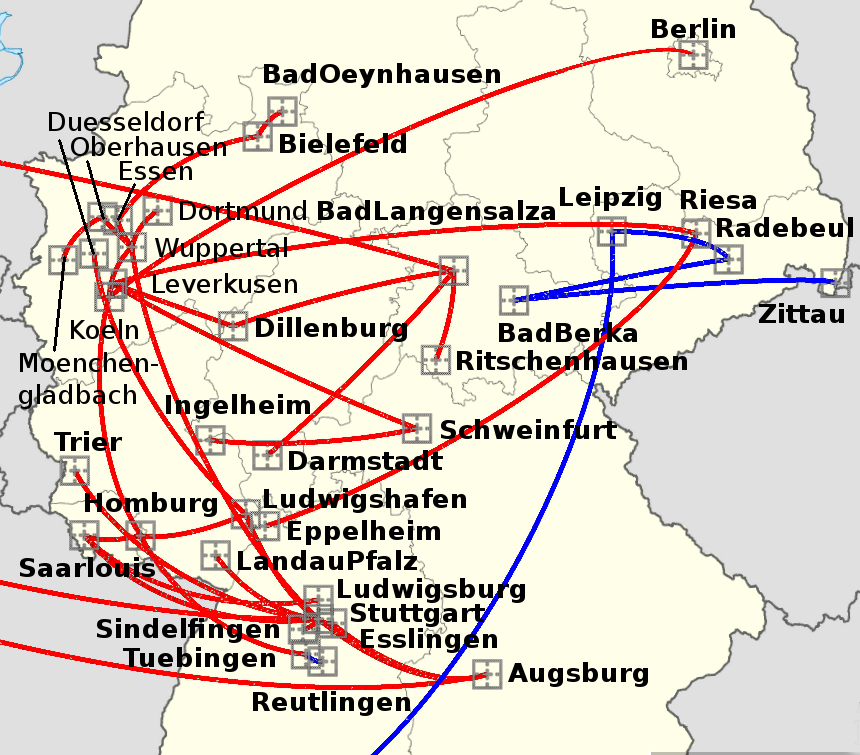

Supplement: S3 Fig — Discrete phylogeographic analysis was performed with BEAST 2.0 and SPREAD 1.0.6. (TIF) [file pone.0139811.s003.tif]
